# 25.1 OLIGODENDROCYTE PATHOLOGY IN PREFRONTAL WHITE MATTER IN SCHIZOPHRENIA

**DOI:** 10.1093/schbul/sby014.101

**Published:** 2018-04-01

**Authors:** Natalya Uranova, Olga Vikhreva, Valentina Rakhmanova, Diana Orlovskaya

**Affiliations:** Mental Health Research Center

## Abstract

**Background:**

Recent neuroimaging studies have shown altered brain connectivity in patients with schizophrenia, associated with disturbed myelination in different fiber tracts and disruptions of white matter (WM) integrity, including prefrontal WM. We aimed to perform a qualitative and morphometric study of the ultrastructure of oligodendrocytes, myelin-forming cells, in prefrontal WM in schizophrenia and normal controls.

**Methods:**

WM of the prefrontal cortex (Brodmann’s area 10) was studied by transmission electron microscopy and morphometry. Size, volume density (Vv) and the number (N) of organelles in oligodendrocytes were estimated in 21 patients with schizophrenia and 20 normal matched controls. Pearson correlation analysis was performed to assess possible correlations between the parameters measured and age, post-mortem interval, neuroleptic treatment and duration of the disease. ANCOVA tests were used for group comparisons.

**Results:**

Qualitative study showed swelling, vacuolation, paucity of ribosomes and mitochondria and accumulation of lipofuscin granules in oligodendrocytes in schizophrenia as compared to controls. Morphometry detected lowered Vv and N of mitochondria and higher Vv and N of lipofuscin granules and vacuoles in oligodendrocytes in the schizophrenic group as compared to the control group (all p<0.01).

**Discussion:**

Altered metabolism of oligodendrocytes, previously reported reduced number of oligodendrocytes, disrupted myelin/axon integrity, damage and progressive degeneration of myelin sheaths in prefrontal WM in schizophrenia may lead to disturbances in myelination, deficiency of nerve impulses propagation and contribute to network dysfunctions in schizophrenia. Oligodendrocyte and myelin abnormalities may be a target to prevent or restore WM abnormalities and dysfunction of neuronal connectivity in schizophrenia.

